# Supplementary material for: Perceived Feasibility of Endoscopic Ultrasound-Guided Gastroenteric Anastomosis: An Italian Survey
Source: Medicina (Kaunas). 2022 Apr 12;58(4):532. doi: 10.3390/medicina58040532 (PMC9027201; doi:10.3390/medicina58040532)
Supplement: Supplementary file 1 [file medicina-58-00532-s001.zip › medicina-1652812-supplementary.pdf]

CHERRIES CHECKLIST

Adapted from:
Eysenbach G. Improving the quality of Web surveys: the Checklist for Reporting Results of Internet E-Surveys (CHERRIES) [published correction appears in doi:10.2196/jmir.2042]. *J Med Internet Res*. 2004;6(3):e34. Published 2004 Sep 29. doi:10.2196/jmir.6.3.e34

DESIGN

DESIGN: The target population is represented by a working group of endoscopists skilled (at least 100 EUS performed individually) in interventional EUS and LAMS placement, from 40 Italian Centers. It reprersents a convenience sample

IRB

IRB: **:** Ethical review and approval were waived for this study, due to the fact that this was only a survey on working group of endoscopists skilled in interventional EUS and LAMS placement, from 40 Italian Centers.

DATA information: The patients were informed via a web app about the purpose of the study

DATA PROTECTION: No personal information were stored

DEVELOPMENT AND PRE-TESTING

DEVELOPMENT AND PRE-TESTING: The aim of this survey is to investigate the perceived feasibility of this procedure nationwide, with the purpose to be helpful in understanding practice patterns, identifying areas of controversies to guide future research towards a beneficial standardization. The rationale for developing the present study was to focus about the perceived feasibility and safety of EUS-GEA in all its indications, both in oncological setting and in benign diseases.

RECRUITMENT PROCESS

OPEN vs CLOSE survey: CLOSE survey, since the patients were contacted via e-mail personally and only the participants belonging to the community of skilled endoscopist could participate

CONTACT MODE: Questionnaires were sent via a web app called I-EUS

ADVERTISING THE SURVEY : advertisement was used to influence participants via e-mail and a web app called I-EUS

SURVEY ADMINISTRATION

WEB/EMAIL: e-survey was sent via a web-app called I-EUS

CONTEXT: endoscopists skilled in interventional endosonography

MANDATORY/VOLUNTARY: Our survey was voluntary

INCENTIVES: no incentives were offered

TIME/DATE: from November 2019 to February 2020

RANDOMIZATION OF ITEMS: N/A

ADAPTATIVE QUESTIONING: Yes

NUMBER OF ITEMS: 49

NUMBER OF SCREEN: 14

COMPLETENESS CHECK: N/A

REVIEW STEP: N/A

RESPONSE RATES

UNIQUE SITE VISITOR: N/A

VIEW RATE: N/A

PARTICIPATION RATE: 29/60 (49%)

COMPLETION RATE: all the endoscopists interviewed completed the questionnaires (100%)

PREVENTING MULTIPLE ENTRIES FROM THE SAME INDIVIDUAL

COOKIES USED: N/A

IP CHECK: N/A

LOG FILE ANALYSIS: N/A

REGISTRATION: N/A

ANALYSIS

HANDLING OF INCOMPLETE QUESTIONNAIRES: only complete questionnaires were analyzed

QUESTIONNAIRE SUBMITTED WITH AN ATYPICAL TIMESTAMP: N/A

STATISTICAL CORRECTION: NO STATISTICAL CORRECTION WAS NEEDED
